# Five-year follow-up of patients with low-risk papillary thyroid cancer treated without postoperative radioiodine: prospective study by the Finnish Thyroid Cancer Group

**DOI:** 10.2340/1651-226X.2025.44458

**Published:** 2025-11-18

**Authors:** Päivi Halonen, Miika Salo, Veera Ahtiainen, Niina Matikainen, Hanna Aula, Johanna Ruohola, Leena Moilanen, Minna Koivikko, Saara Metso, Emmi Peurala, Hanna Mäenpää, Finnish Thyroid Cancer Group

**Affiliations:** a Department of Oncology, Comprehensive Cancer Center, Helsinki University Hospital and Helsinki University, Helsinki, Finland; b Helsinki University, Helsinki, Finland; c Endocrinology, Abdominal Center, Helsinki University Hospital and University of Helsinki, Helsinki, Finland; d Department of Oncology, Tampere University Hospital, Tampere, Finland; e Department of Oncology, Turku University Hospital, Turku, Finland; f Endocrinology and Clinical Nutrition, Department of Medicine, Kuopio University Hospital, Kuopio, Finland; g Department of Internal Medicine, Oulu University Hospital, and Oulu University, Oulu, Finland; h Department of Internal Medicine, Endocrinology, Tampere University Hospital, Wellbeing Services County of Pirkanmaa and Faculty of Medicine and Health Technology, Tampere University, Tampere, Finland

**Keywords:** Low-risk thyroid carcinoma, neck ultrasound, radioiodine treatment, thyroglobulin, thyroglobulin antibodies

## Abstract

**Background and purpose:**

The purpose of this study was to evaluate the safety of omitting radioiodine (RAI) ablation in low-risk papillary thyroid cancer.

**Patients and methods:**

All five university hospitals in Finland consecutively and prospectively enrolled patients in the study with the following inclusion criteria: age 18 or over, papillary unifocal, intrathyroidal cancer 11–20 mm operated with a thyroidectomy, and no lymph node metastases. All patients were initially offered a follow-up without RAI. The patients who did not receive postoperative RAI were included in the RAILESS group. Those who preferred to have RAI and those who received RAI due to elevated thyroglobulin (TG) or thyroglobulin antibodies (TGAb) formed the RAIRINN group. Thyroglobulin and TGAb levels were monitored 4–8 weeks postoperatively in the RAILESS group. All patients were subsequently monitored every 3 months for the first year and then annually for 5 years, with a neck ultrasound. Radioiodine was administered if TG surpassed 2 ug/L or TGAb exceeded 40 kU/L in two consecutive measurements. An event was defined as a structural recurrence or a biochemical abnormality resulting in RAI treatment. The primary endpoint was the amount of patients who remained event-free during a 5-year follow-up.

**Results:**

Fifty-three of 60 patients enrolled were assigned to the RAILESS and 5 to the RAIRINN group. In the RAILESS group, 96% (51/53) remained event-free throughout 5 years, while 4% (2/53) required RAI due to increased TG or TGAb levels. In the RAIRINN group, one patient (1/7 or 14%) developed a metastatic disease.

**Interpretation:**

Our findings provide additional evidence for safely omitting postoperative RAI in low-risk papillary thyroid cancer.

## Introduction

The primary therapy goals in the treatment of differentiated thyroid cancer (DTC) are to improve overall and disease-specific survival and to reduce the risk of persistent and recurrent disease and morbidity. Accurate disease staging and risk stratification are crucial in minimising treatment-related morbidity and unnecessary therapy [1]. Until recently, it was standard practice to administer radioiodine (RAI) therapy after thyroidectomy to all patients with DTC, although the benefits of radioiodine administration in low-risk thyroid cancer have been controversial [1, 2].

The benefits of forgoing routine postoperative RAI for patients with low-risk thyroid cancer are numerous. Radiation exposure, adverse effects, sick leaves from work, and hospitalisation can be avoided without any radioiodine treatment. The costs will be lower, and there will be no RAI -induced sialadenitis, which has a major impact on patients’ quality of life [3–5].

Retrospective studies have not shown the survival advantage of postoperative administration of RAI in patients with low-risk DTC, and the results in terms of recurrence have been conflicting or uncertain [6]. Two endpoints have been used for the prognostic classifications of DTC. The American Thyroid Association (ATA) risk stratification predicts the risk of structural recurrence and the TNM classification the risk of cancer-related death [1, 6, 7].

In over 90% of patients, the risk of cancer-related death is less than 2% [6]. The risk of persistent or recurrent disease is much higher than the risk of cancer-related death, suggesting that most recurrences can be cured or at least controlled in the long term, especially in patients with low-risk DTC. The ATA has defined three groups of patients with different risks of recurrence: low (< 5%), intermediate (5–20%), or high risk (> 20%) [1, 8]. Over 80% of patients with DTCs are classified as low-risk.

Patients in any risk group older than 55 years carry an increased risk of recurrence and a lower probability of cure after recurrence [9, 10]. The ATA risk stratification does not consider age as a risk factor for structural recurrence [1]. Meanwhile, in the TNM classification for risk of cancer-related death the age of 55 years is a cut-off point for a greater risk of death [7, 11].

Recently, two large prospective randomised phase 3 trials supporting a follow-up in lieu of RAI ablation in low-risk DTC have been published. The first of them, the ESTIMABL2 trial published in 2022 by Leboulleux et al., showed non-inferiority of the follow-up strategy without any RAI compared to RAI ablation in patients with low-risk thyroid cancer who had undergone thyroidectomy at the 3-year timepoint [12]. The results of another prospective randomised non-inferiority trial (IoN) comparing the outcome in patients with low-risk DTC treated either with or without RAI of 1.1 GBq were recently published [13]. In this study, no difference was found in the 5-year recurrence-free rates between RAI ablation and non-ablation groups indicating that non-inferiority was reached.

At the same time that two forementioned large randomised prospective trials were launched to investigate the non-inferiority of omitting RAI versus administering RAI in low-risk thyroid cancer, the Finnish Thyroid Group also initiated a prospective national study on this issue. The objective was to investigate the 5-year outcomes in patients diagnosed with low-risk papillary thyroid cancer who did not receive RAI therapy. Those patients who received radioiodine therapy according to standard practice were concurrently monitored. Overall, the purpose of the study was to evaluate the safety of omitting RAI in patients with low-risk papillary thyroid cancer in Finland.

## Patients and methods

### Trial oversight and ethics

The study was a prospective, non-randomised, open-label Finnish national study planned, designed, and performed by the Finnish Thyroid Cancer Group. Patients were consecutively enrolled in the study at all five university hospitals in Finland (Helsinki, Kuopio, Oulu, Tampere and Turku) from October 2014 to November 2017.

The study received approval from the Central Ethics Committee of Helsinki University Hospital, allowing it to be conducted in other university hospitals in Finland as well. It was conducted in accordance with the Declaration of Helsinki and the International Conference on Harmonization on Good Clinical Practices. All patients provided written informed consent before enrolling in the trial. The trial, named RAILESS, was registered with ClinicalTrials.gov under the document number NCT06271044.

### Primary endpoint

The primary endpoint was the number of patients who remained event-free during a 5-year follow-up period. An event was defined as either a structural recurrence requiring treatment or a biochemical abnormality determined as an increase in TG or TGAb levels, as directed by the study protocol, resulting in RAI. The outcome was evaluated at 3, 4, and 5-year postoperative intervals. Moreover, data was collected on TG and TGAb levels in study patients during the follow-up period.

### Patients, methods, and procedures

The inclusion criteria were age 18 or over, unifocal and intrathyroidal papillary cancer with a diameter of 11–20 mm, thyroidectomy as the surgical treatment, and no known radiological or histopathological lymph node metastases (Nx or N0). Patients with a pregnancy, or operated with a lymphadenectomy alongside a thyroidectomy, or with any aggressive characteristics in the papillary tumour upon histopathological examination were excluded.

The study patients, who fulfilled the inclusion criteria and gave their consent to participate, did not receive a postoperative radioiodine treatment but were systematically followed up for at least 5 years after the thyroidectomy. This group was referred to as the RAILESS group.

As it was standard practice to administer a radioiodine treatment to low-risk patients at the time of the study, patients who preferred RAI over a follow-up without radioiodine were given the treatment with an activity of 1.1 GBq following the stimulation with recombinant human thyroid-stimulating hormone (rhTSH), and asked to be followed up for at least 5 years. These patients formed the RAIRINN group. Based on the study protocol, the serum TG level was measured in the RAILESS group 4–8 weeks postoperatively. For values between 2 to 5 µg/L, a subsequent measurement was scheduled 3 months postoperatively. The RAIRINN group also included patients with a TG level elevated up to 2 µg/L or higher at the 3-month mark, who therefore received RAI with an activity of 1.1 GBq.

The levels of serum TG and TGAb were measured every 3 months during the first year of the follow-up period on levothyroxine treatment, and thereafter annually until 5 years. The measurements of a stimulated TG were not routinely performed in any of the study patients in the RAILESS group during a follow-up. In the RAIRINN group a stimulated TG was measured 8–12 months after RAI therapy according to our Finnish follow-up protocol. An ultrasound of the neck was performed annually for 5 years after the operation.

**Figure 1:**
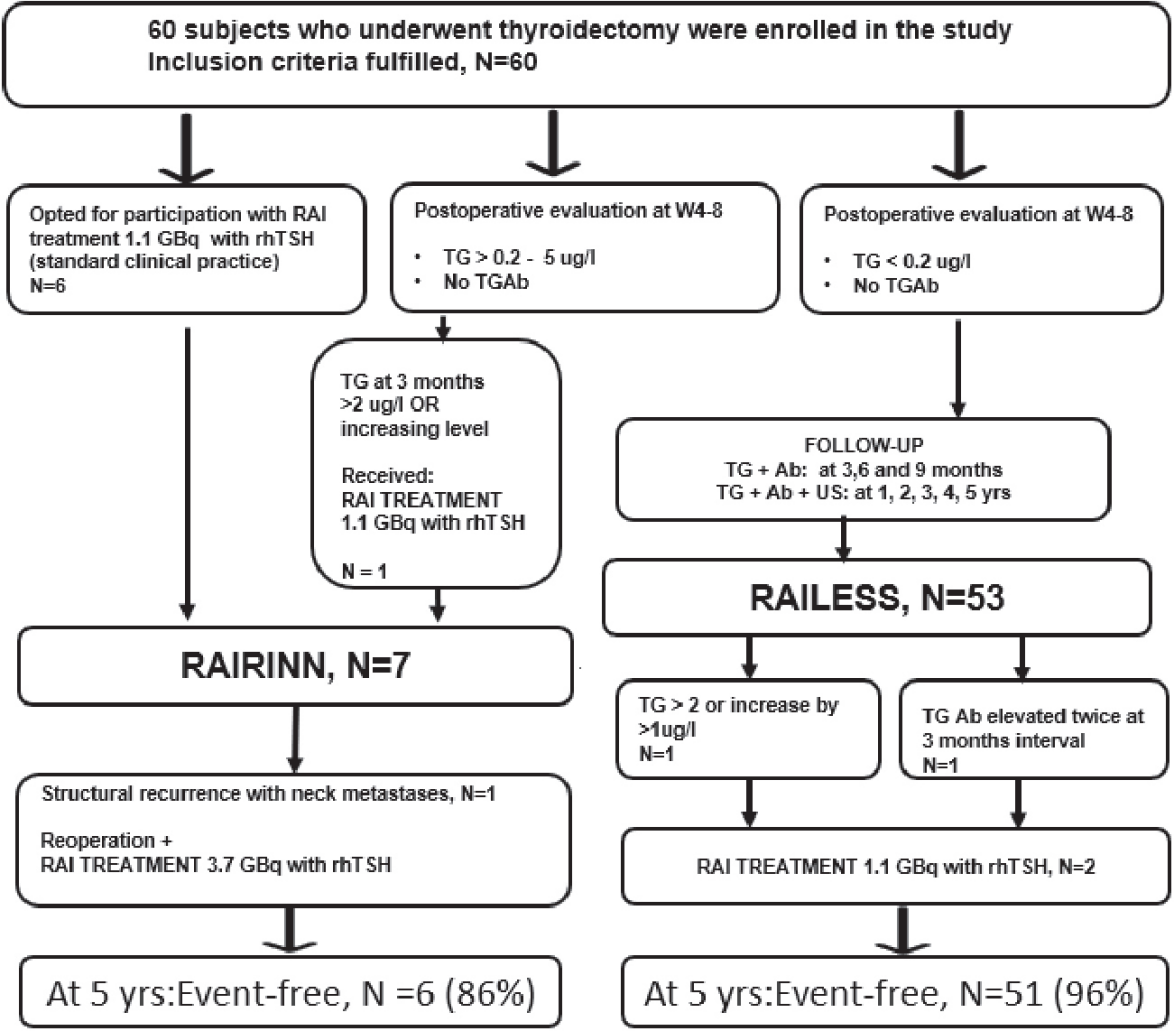
The enrolment, follow-up schedule and 5-year outcome of the study patients. The RAILESS group consisted of 53 patients with no postoperative RAI. Seven patients received RAI and formed the RAIRINN group. Six of them underwent RAI postoperatively following standard practice, and one because the TG level exceeded 2 µg/L at the 3-month follow-up point.

In cases where a rise in serum TG was observed, reaching 1 µg/L or higher compared to the lowest level recorded during the study, the study protocol mandated a radioiodine treatment of 1.1 GBq with rhTSH even though there were no signs of structural recurrence or metastases. Additionally, radioiodine treatment was administered if the TGAb level exceeded 40 kU/L and remained elevated in two consecutive measurements taken 3 months apart. If multiple RAI treatments were required, an activity of 3.7 GBq was chosen (Figure 1).

TG levels were assessed using either a commercial chemiluminescence immunoassay (Siemens Healthineers Immulite Xpi 2000 analyzer) or an electrochemiluminescence immunoassay (Roche Diagnostics Cobas analyzer). TGAb levels, on the other hand, were determined through fluorescence enzyme immunoassay.

### Statistics

The statistical analyses were performed using the Statistical Package for the Social Sciences (SPSS) software version 29.00 (SPSS Inc., Chicago, IL, United States). Descriptive quantitative data were expressed as medians and ranges.

## Results

The follow-up period concluded on April 1, 2023, with 60 patients initially included in the study. Figure 1 illustrates the enrolment, follow-up schedule and outcome of the study patients in the RAILESS and RAIRINN groups.

Among the participants, the majority were women, accounting for 85% (51/60), with a median age of 51 years (range 18 to 80 years). Table 1 presents the study patients’ age and gender along with the main results. Table 2 displays the TG and TGAb levels, as well as the neck ultrasound findings recorded at the 3, 4, and 5-year follow-up intervals.

**Table 1:** The age and gender of the study patients and the main results of the study.

VARIABLE	RAILESS	RAIRINN
	(*n* = 53)	(*n* = 7)
Age, years, median (range)	53 (18–73)	57 (33–80)
Gender, *n* (%)		
Female	45 (85%)	5 (71%)
Male	8 (15%)	2 (29%)
Event-free patients, *n* (%)	51 (96%)	6 (86%)
Events during follow-up, *n* (%)	2 (4%)	1 (14%)
Biochemical abnormality		
resulting in RAI treatment		
RAI due to TG elevation	1 (2%)	1 (14%)
RAI due to TGAb elevation	1 (2%)	0 (0%)
Structural recurrence	0 (0%)	1 (14%)

**Table 2:** Levels of thyroglobulin and thyroglobulin antibodies and neck ultrasound (US) findings after the follow-up of 3, 4 and 5 years.

VARIABLE	RAILESS	RAIRINN
	(*n* = 53)	(*n* = 7)
TG (ug/L), median and range		
at 3 years	0.0 (0–2.7)	0.0 (0–0)
at 4 years	0.0 (0–1.9)	0.0 (0–0)
at 5 years	0.0 (0–1.7)	0.0 (0–0)
TGAb (kU/L), median and range		
at 3 years	0.0 (0–136.4)	0.0 (0–42)
at 4 years	0.0 (0–132.4)	0.0 (0–67)
at 5 years	0.0 (0–166.0)	0.0 (0–58)
TG (ug/L), *n* (%) not measurable / 0.1– < 2 / ≥ 2 / missing		
at 3 years	36 (68%) / 15 (28%) / 1 (2%) / 1 (2%)	7 (100%) / 0 / 0
at 4 years	39 (74%) / 12 (23%) / 0 / 2 (4%)	5 (71%) / 0 / 0 / 2 (29%)
at 5 years	39 (74%) / 12 (23%) / 0 / 2 (4%)	6 (86%) / 0 / 1 (14%)
TGAb levels (kU/L), *n* (%) not elevated / elevated (> 40 kU/L) / missing		
at 3 years	51 (96%) / 1 (2%) / 1 (2%)	7 (100%) / 0 / 0
at 4 years	50 (94%) / 1 (2%) / 2 (4%)	5 (71%) / 0 / 2 (29%)
at 5 years	50 (94%) / 1 (2%) / 2 (4%)	6 (86%) / 0 / 1 (14%)
Neck US findings, *n* (%) no evidence of disease / needs follow-up / missing		
at 3 years	47 (89%) / 5 (9%) / 1 (2%)	7 (100%) / 0 / 0
at 4 years	45 (85%) / 3 (6%) / 5 (9%)	6 (86%) / 0 / 1 (14%)
at 5 years	48 (91%) / 1 (2%) / 4 (8%)	6 (86) / 0 / 1 (14%)

Out of 53 patients, 51 (96%) in the RAILESS group remained event-free during the 5-year follow-up period. Two patients (4%) received RAI during the follow-up period. Of these two receiving RAI, one was due to a gradual increase in TG level up to 3 µg/L, while the other was attributed to a rise in TGAb level over 40 kU/L, confirmed in two consecutive measurements taken 3 months apart. Notably, neither of them showed evidence of structural recurrence or metastases. These events occurred within the first year post-operation, with RAI administered 11 and 7 months after their initial surgeries, respectively. Furthermore, during the trajectory of the follow-up period, one of the patients who received radioiodine treatment was found to have heterophilic antibody interference in further laboratory analyses, which resulted in a false positive TG level. After the removal of heterophilic antibodies, the TG level was < 0.1 µg/l. One of the seven patients (1/7), or 14% in the RAIRINN group, was diagnosed with locoregional lymph node metastases 1 year after the primary operation. Retreatment involved a neck dissection to remove metastatic lymph nodes, followed by RAI with an activity of 3.7 GBq.

## Discussion and conclusions

Our study demonstrated favourable outcomes over a 5-year follow-up period for patients with low-risk papillary cancer who underwent thyroidectomy without routine postoperative RAI.

In the RAILESS group, 96% of patients remained event-free throughout the entire 5-year follow-up period. Only 4% received delayed RAI due to elevated TG or TGAb levels. None of the patients had structural recurrence. Notably, the sole patient experiencing structural recurrence was classified in the RAIRINN group. Furthermore, one patient who received RAI subsequently revealed a false positive TG from heterophilic antibody interference that was not connected with thyroid cancer.

Our study findings align with the results of two recent prospective, randomised, phase 3 trials investigating, whether a follow-up without RAI is non-inferior compared to RAI ablation after a thyroidectomy in patients with low-risk DTC. The largest of these, the 2022 ESTIMABL2 trial by Leboulleux et al. [12], reported the outcome after a 3-year follow-up period. The IoN trial by Mallick et al. was recently published, presenting the 5-year recurrence-free rates for study population [13].

In the ESTIMABL2 trial, the investigators assigned patients with low-risk DTC after a thyroidectomy to either receive ablation with a postoperative administration of radioiodine (1.1 GBq) after injections of rhTSH (radioiodine group) or receive no postoperative radioiodine (no-radioiodine group) [12]. In contrast to our study, the ESTIMABL2 study included not only papillary but also follicular and oncocytic cancer, with a multifocal pT1a tumour (a diameter of each lesion ≤ 1 cm and a sum of the longest diameters of the lesions ≤ 2 cm) or a pT1b tumour (> 1 cm and ≤ 2 cm). In addition, a neck dissection was performed in 44% of the study patients. The primary objective was to assess whether the omission of radioiodine therapy was non-inferior to radioiodine therapy in terms of the absence of a composite endpoint that included functional, structural, and biological abnormalities at the 3-year mark. Non-inferiority was defined as a between-group difference of less than 5 percentage points in the proportion of event-free patients. The ESTIMABL2 trial included 730 patients who could be evaluated 3 years after randomisation. Among them, the proportion of patients without an event was 95.6% in the no-radioiodine group and 95.9% in the radioiodine group. The results met the non-inferiority criteria. The study events consisted of structural or functional abnormalities in eight patients and biological abnormalities in 23 patients resulting in a total of 31 events. Events occurred more frequently in patients with a postoperative serum TG level of more than 1 ng/ml during the thyroid hormone treatment [12]. Furthermore**,** the non-inferiority of a follow-up strategy compared with postoperative RAI administration in low risk DTC in the ESTIMABL2 trial was confirmed at 5 years. Of the enrolled patients 698 were evaluable at 5 years. The proportions of patients without events were 93.2% in the no-radioiodine group and 94.8% in the radioiodine group, for a difference of −1.6% [14].

The most recent multicentre IoN trial by Mallick et al. [13] was designed to assess whether recurrence-free survival was non-inferior after no ablation compared with ablation in patients with low-risk DTC. Their study encompassed 504 patients, recruited at 33 UK cancer centres, who had a complete (R0) resection following the total thyroidectomy; stage pT1, pT2, pT3 (according to TNM7), or pT3a (according to TNM8) disease; and N0, Nx, or N1a disease. Participants were randomly assigned 1:1 to receive either 1.1 GBq ablation or no ablation, following the thyroidectomy. The 5-year recurrence-free rates were 97.9% in the no ablation group versus 96.3% in the ablation group in the ITT analysis, and 97.9% versus 96.9% in the per-protocol analysis. The 5-year absolute risk difference was 0.5 percentage points showing that non-inferiority was reached [13]. Unlike both the ESTIMALB2 trial and our study, which recruited patients with pT1 thyroid cancers, the IoN trial included patients with pT2 and pT3 papillary and follicular cancer, even if tumours with aggressive pathological features were excluded. Despite the inclusion of patients with more advanced disease and follicular thyroid cancer the IoN study surprisingly showed a similarly high proportion of event-free patients as our study [13].

In a prospective, multicentre, non-randomised study, Ilera et al. [15] recruited 174 patients with a low risk of recurrence DTC according to the modified 2015 ATA risk stratification system; all participants had undergone a thyroidectomy and were divided into two groups. One group consisted of 87 patients who received RAI, while the other group of 87 patients did not. The evaluation of treatment responses occurred 6–18 months post-thyroidectomy and at the end of the follow-up period. This evaluation involved the measurements of TG and TGAb levels and neck ultrasonography. The initial treatment response was comparable between the two groups, with < 2% showing a structural incomplete response. The final status was assessed in 139 cases, with a median follow-up duration of 60 months, surpassing that of the trial conducted by Leboulleux et al. [12]. Among patients who received RAI, 82.8% exhibited no evidence of disease (NED), 12% had an indeterminate response (IR) and 5% a biochemical incomplete response (BIR). Conversely, among patients who did not undergo RAI, 90% had NED, 8.7% had IR, and 1.2% had BIR. No statistical difference was found between the two groups. Furthermore, no patient had evidence of a structural disease at the end of follow-up period [15]. These results also align with our findings.

Janovsky et al. [16] have also reported the results of a prospective follow-up study in which 57 patients with low-risk thyroid cancer were treated with a thyroidectomy and without postoperative RAI. They were followed up for 36–84 months without any sign of recurrence. Their study did not include any patients receiving postoperative RAI [16].

We acknowledge several limitations in our study. Firstly, we were unable to utilise the response evaluation system outlined in the ATA 2015 classification since our study protocol was designed and initiated in October 2014, prior to the publication of the ATA guidelines in 2016. Additionally, our study suffers from a relatively small sample size. It was also conducted without randomisation, instead opting for the open-label recruitment of consecutive patients. Moreover, the intended two study groups, RAILESS and RAIRINN, were not evenly balanced in terms of size. The RAIRINN group ultimately comprised only seven patients, rendering the comparisons between these groups impractical. Furthermore, our study focused solely on patients with low-risk pT1 unifocal papillary thyroid cancer, excluding those with low-risk follicular thyroid cancer.

For years, thyroid cancer management has primarily relied on retrospective studies, which may introduce biases. Recently, two large randomised trials have shown non-inferiority of the follow-up strategy without any RAI compared to RAI ablation after a thyroidectomy in low-risk DTC after 3-year [12] and 5-year [13] follow-up period. Overall, our study also contributes to the field of thyroid cancer management, as it provides evidence supporting the safe omission of routine postoperative radioiodine treatment in patients with low-risk papillary thyroid cancer.

## Data Availability

Data used for the paper can be found at the Cancer Center, Department of Oncology, Helsinki University Hospital and accessed by the corresponding author.
